# Green Synthesis and Characteristics of Cellulose Nanocrystal/Poly Acrylic Acid Nanocomposite Thin Film for Organic Dye Adsorption during Water Treatment

**DOI:** 10.3390/polym15092154

**Published:** 2023-04-30

**Authors:** Amani Saleh Almuslem, Nisrin Alnaim, Sobhy S. Ibrahim, Mostafa A. Ibrahim

**Affiliations:** 1 Physics Department, College of Science, King Faisal University, Al Ahsa 31982, Saudi Arabia; nalnaim@kfu.edu.sa; 2 Physics Department, Faculty of Science, Cairo University, Giza 12613, Egypt; ssibrahim@sci.cu.edu.eg; 3 Chemistry Department, Faculty of Science, Helwan University, Cairo 11795, Egypt; mustafaahmednano@gmail.com; 4 Production and R&D Unit, NanoFab Technology Company, 6th October City, Giza 11795, Egypt

**Keywords:** cellulose nanocrystal, poly acrylic acid, active carbon, polymers, crosslinked membranes, nanocomposites, organic pollutants, methylene blue, adsorption parameters

## Abstract

Nanocellulose shows potential as an effective natural adsorbent for removing harmful contaminants from wastewater. This paper describes the development of innovative nanocellulose thin films made of cellulose nanocrystals (CNCs), polyacrylic acid (PAA), and active carbon (AC) as adsorbent materials for absorbing azo dyes from wastewater. The CNCs were recovered from sugarcane bagasse using alkali treatment and acid hydrolysis. The composition and processing parameters of the thin films were optimized, and their adsorption capacity was determined using thermodynamic isotherms and adsorption kinetics. Adsorption characteristics such as the methylene blue (MB) dye concentration, contact time, temperature, and pH were investigated to determine how they affected adsorption. The results show that the adsorption process follows pseudo-second-order kinetics. At an adsorbent mass of 50 mg, dye concentration of 50 ppm in 50 mL, and contact period of 120 min at 25 °C, the thin film comprising 64 wt% CNC, 16 wt% PAA, and 20 wt% AC showed high dye removal efficiency (86.3%) and adsorption capacity (43.15 mg/g). The MB removal efficiency increased to 95.56% and the adsorption capacity to 47.78 mg/g when the medium’s pH was gradually increased from neutral to alkaline. The nontoxicity, low production cost, water stability, easy recovery, and high adsorption capacity of these membranes make them suitable for water treatment systems.

## 1. Introduction

The presence of dyes in effluents is a major problem due to the negative effects they have on many different types of life. Dye discharge into the environment is a source of concern for both toxicological and aesthetic reasons [1]. Textile, leather, paper, plastics, and other industries utilising dyes consume large amounts of water. As a result, they produce a significant volume of coloured wastewater [2]. More than 100,000 commercially accessible dyes are expected to be available, with over 7 × 105 tonnes of dyestuff produced annually [3,4,5]. It is widely acknowledged that the colour of water has a significant impact on public impressions of its quality. Colour is the first pollutant found in wastewater. Even trace levels of dyes in water—less than 1 ppm for some dyes—are highly visible and unwanted [6,7]. MB is the most commonly used dyeing agent for cotton, wood, and silk. It can induce eye burns, which can result in irreversible damage to human and animal eyes. It can cause short periods of fast or difficult breathing when inhaled, whereas absorption through the mouth causes a burning feeling, nausea, vomiting, intense perspiration, mental confusion, and methemoglobinemia [8,9,10]. Because of the negative effects on the receiving waterways, the treatment of wastewater containing such dye is of importance.

Many physical, chemical, and biological decolorization procedures have been published during the last three decades; however, few have been approved by the paper and textile industries [11]. Of the several dye removal processes, adsorption is the procedure of choice and produces the greatest results because it can be used to remove various types of colouring chemicals [12,13,14]. Many ways have recently been investigated for the production of less expensive and more effective adsorbents. Several researchers have proposed a variety of non-traditional low-cost adsorbents, including natural materials, biosorbents, and agricultural and industrial waste materials. These materials could be utilised as dye adsorbents to remove dyes from solutions. Many treatment processes have been applied for the removal of dyes from wastewater, such as photocatalytic degradation [15], sonochemical degradation [16], micellar enhanced ultrafiltration [17], cation exchange membranes [18], electrochemical degradation [19], adsorption/precipitation processes [20], integrated chemical–biological degradation [21], integrated iron(III) photo-assisted-biological treatment [22], solar photo-Fenton and biological processes [23], Fenton-biological treatment schemes [24], and adsorption on activated carbon [25]. Because synthetic dyes in wastewater cannot be efficiently decoloured using standard methods, the adsorption of synthetic dyes on affordable and efficient solid supports was proposed as a simple and cost-effective method for their removal from water and wastewater [26]. Dye wastewater treatment methods have been reviewed. Adsorption is a well-known equilibrium separation procedure that is excellent for water purification [27,28]. In terms of initial cost, flexibility and simplicity of design, ease of operation, and sensitivity to harmful contaminants, adsorption has been proven to be superior to other systems for water re-use.

This study describes the development of a thin film consisting of natural components (organic (CNC and PAA) and inorganic (AC) adsorbents) to remove cationic dye. These thin films are suitable for water treatment due to their nontoxicity, high adsorption capacity with a short contact time, and mechanical stability in the water system. Incidentally, the CNCs were derived from sugarcane bagasse, adding to agricultural waste recycling. This study used methylene blue (MB) as a model for a cationic-soluble azo dye. Its toxicity, carcinogenicity, and resistance to biodegradation pose major risks to human health and the environment [29]. The thin film’s composition and adsorption parameters (thin film mass, initial dye concentration, initial pH, temperature, and contact time) were optimized. Its adsorption capability was assessed using thermodynamic and kinetic experiments. AC is an effective material for dye removal [30] due to its high specific surface area, well-defined pores in molecular dimensions, outstanding physical and chemical stability, and significant sorption capability for cationic dyes [31]. PAA’s high surface functionality (abundant carboxyl groups) can improve the cationic dye adsorption ability of the produced thin film [32]. Recently, the application of sustainable nanomaterials such as cellulose nanocrystals (CNCs) for dye adsorption from water was reported [33], as well as pollutant adsorption from wastewater [34]. For example, cellulose acetate has been used to create adsorbents (particles, films, and membranes) for the removal of wastewater dyes [35]. Cellulose nanocrystals (CNCs) are typically made from cellulose present in a wide range of wood, bacteria, algae, tunicates, and other organisms [36]. Agricultural wastes such as rice straws and bagasse can also be used to make CNCs [37], which can be used in a variety of water treatment applications [38]. A popular method for creating CNCs is acid hydrolysis using HCl or H_2_SO_4_. H_2_SO_4_ interacts with the OH groups on the surface of the cellulose to generate sulphate half esters, which result in negatively charged, electrostatically stabilised CNCs [39]. After acid treatment with HCl, the surface OH groups remain unaltered, resulting in CNCs with a low surface charge density and a high flocculation potential. CNCs’ negatively charged groups (–OH, –SO_3_, -COO) attract cationic dyes electrostatically [40]. CNCs can also be chemically modified to improve their affinity for anionic and nonionic dyes. A novel adsorbent for the removal of dyes from various contaminated water can be created using graft polymerization and the coupling of PAA and CNCs with inorganic adsorbents (e.g., clay, zeolite) and carbon (active carbon, graphene, carbon nanotubes) [41,42].

Our work showed that CNC/PAA/AC thin films with tunable CNC/PAA compositions can improve MB’s adsorption behaviour. Their adsorption activities, including kinetics, isotherms, thermodynamics, and pH effects, were thoroughly examined. Several mathematical models were used to evaluate and analyse the kinetics and isotherms of the adsorption process to explain the interaction mechanisms between MB and thin films. Finally, desorption tests were used to assess the thin film’s reusability.

## 2. Experimental Procedures

### 2.1. Materials

Sugarcane bagasse was produced in Cairo, Egypt. CNCs were synthesized using HCl (37%; Merck, Lebanon, NJ, USA), sodium hydroxide (98%; Daejung, Sihung, Korea), hydrogen peroxide (H_2_O_2_; 85 wt% in water; Sigma-Aldrich, St. Louis, MO, USA), sodium hypochlorite (NaOCl; 13%; Chem-Lab NV, Zedelgem, Belgium), and H_2_SO_4_ (97%; Emparta, Taufkirchen, Germany). MB (C_16_H_18_ClN_3_S; El-Nasr Company, Cairo, Egypt), citric acid (C_6_H_8_O_7_; 99%; Sigma-Aldrich), AC (99%; Nano Fab Technology, Giza, Egypt), and MilliQ water were also used in the experiments.

### 2.2. Extraction of Cellulose Nanocrystals (CNCs) from Sugarcane Bagasse

CNC synthesis was carried out in many stages. Initially, sugarcane bagasse was sun-dried for 24 h before being cut into small pieces and de-waxed by heating 20 g dry weight in 1000 mL of water to 90 °C for 2 h with constant stirring and repeating the technique twice to thoroughly remove the wax. Second, the powder was then acid treated for 2 h at 60 °C with 400 mL of 10% HCl to remove inorganic minerals. Third, the demineralized powder was washed with water until it reached a neutral pH to remove any leftover acid and minerals. Fourth, 350 mL of 4 *w*/*v*% sodium hydroxide (NaOH) was added to the pulp and agitated for 2 h at 60 °C to remove lignin and hemicellulose. The pulp was then washed in distilled water until the pH reached neutral. Fifth, the pulp was bleached for 2 h at 60 °C with mechanical agitation with 250 mL of 24 *v*/*v*% H_2_O_2_ and then rinsed with distilled water until the pH was neutral. Sixth, the pulp was bleached at 90 °C for 4 h with mechanical stirring with 200 mL of 2 *w*/*v*% NaOCl, then washed, filtered, and dried to yield chemically pure pulp. Lastly, CNCs were created by acid hydrolysing the resulting cellulose fibres for 2 h at 45 °C with vigorous stirring in 180 mL of 50 *v*/*v*% H_2_SO_4_. The CNCs were then rinsed several times until the pH was neutral before being centrifuged for 15 min at 4000 rpm and drying overnight at 50 °C.

### 2.3. Fabrication and Characterization of Thin Film

Five thin films with varying dry weight concentrations of 0–64 wt% CNC, 16–80 wt% PAA, and 0–20 wt% AC were produced (Table 1). The thin film’s solid composition (CNC, PAA, and AC) was adjusted to 1 g in 20 mL of water. First, CNC, PAA, and AC were ultrasonically mixed for 30 min before being magnetically swirled for 60 min. Next, the mixture was agitated for 15 min with a mechanical stirrer before being allowed to sit overnight to remove air bubbles. Then, the mixture was placed onto 9 cm diameter Petri dishes and air-dried for 48 h. Finally, the dried thin film was heated to 140 °C for 30 min to accelerate the reaction between the components (CNC, PAA, and AC) until crosslinking occurred and then washed with deionized water to remove unreacted reagents.

Various approaches were used to characterize the physicochemical parameters of AC, CNCs, and the produced thin films. The CNCs’ crystalline phase was determined by X-ray diffraction (XRD; Bruker AXS D8, Germany). Changes in the functional groups of the CNCs and PAA after crosslinking were assessed using Fourier transform infrared (FTIR) spectrophotometry (JASCO model 6700, USA). The particle size and shape of CNCs and AC particles were examined using transmission electron microscopy (TEM; JEOL-JEM-2100, Japan). The thin films’ surface morphology and elemental composition were examined using field emission scanning electron microscopy (FE-SEM; Japan, EOL JSM-6510LV QSEM, Japan). Thin films (1 × 1 cm) were immersed in 50 mL of water for 24 h at room temperature to assess their swelling properties. The thin films’ dry weight was determined before and after water immersion using the equation: swelling (%) = (W_2_/W_2_ − W_1_) 100), where W_1_ is the dry weight of the thin film, and W_2_ is its swollen weight after immersion.

### 2.4. Adsorption Studies

Batch adsorption studies on the produced thin film were conducted using a UV-Vis spectrophotometer (Perkin Elmer Lambda 41) at 665 nm [43]. The adsorption parameters tested included thin film mass (50 mg), contact time (30–180 min), initial dye concentrations (10–100 ppm), pH (3–12), and temperature (25 °C, 45 °C, and 60 °C). The solution dye volume remained constant at 50 mL, and the pH was changed with diluted NaOH and HCl. The equilibrium adsorption capacity (Q_e_; mg/g) and percentage of MB dye removed were calculated using Equations (1) and (2), respectively:

Q_e_ = (C_0_ − C_e_) V/W (1)

Dye removal (%) = (C_0_ − C_e_) × 100/C (2)

where C_e_ (in mg/L or ppm) is the adsorbate equilibrium concentration, C_0_ (in mg/L or ppm) is the initial MB concentration, V is the volume of the solution (in L; 50 mL dye solution), and W is the weight of the adsorbent (in g; 5 mg).

### 2.5. Adsorption Kinetics and Isotherm

The MB dye’s adsorption capacity was determined as a function of contact time using adsorption kinetics with a thin film mass of 50 mg, initial MB dye concentration of 50 ppm, pH of 7, and ambient temperature of 25 °C. The pseudo-first-order and pseudo-second-order adsorption kinetic models were then used to fit the experimental kinetic data. Equation (3) shows the linear version of the pseudo-first-order kinetic model of adsorption solid–liquid systems:

(3) $$\log\left(q_{e}-q_{t}\right)=\log(q_{e})-\frac{K_{1}}{2.303}t$$

Equation (4) shows the pseudo-second-order kinetic model based on the MB chemisorption hypothesis:

(4) $$\frac{t}{q_{t}}=\frac{t}{q_{e}}+\frac{1}{K_{2}q_{e}^{2}}$$

where *q_t_* (in mg/g) is the adsorption capacity at the given contact time *t*, *q_e_* (in mg/g) is the adsorption capacity at the equilibrium time, and *k*_1_ and *k*_2_ are the rate constants for pseudo-first-order and pseudo-second-order models, respectively.

### 2.6. Thermodynamic Isotherm

The thin film (C_4_) with the best favourability, reversibility, and energy for MB dye adsorption was investigated using adsorption thermodynamics. The Langmuir and Freundlich isotherms were used to study the interactions between the MB dye and the best thin film [44]. Then, the Van’t Hoff equation (Equations (5) and (6)) was used to estimate thermodynamic parameters such as changes in Gibbs free energy (∆G°), enthalpy (∆H°), and entropy (∆S°).

ln K = −∆G°/RT(5)

ln K = −(∆H/RT) + (∆S°/R)(6)

where the equilibrium constant/distribution coefficient K = Q_e_/C_e_, T is the solution’s temperature (in K), and R is the universal gas constant (8.314 J/mol K). The changes in enthalpy (∆H°) and entropy (∆S°) were calculated from the slope and intercept of the ln(K) vs. 1/T plot, respectively.

## 3. Results and Discussion

### 3.1. Characteristics of CNCs and AC Particles

**Figure 1:**
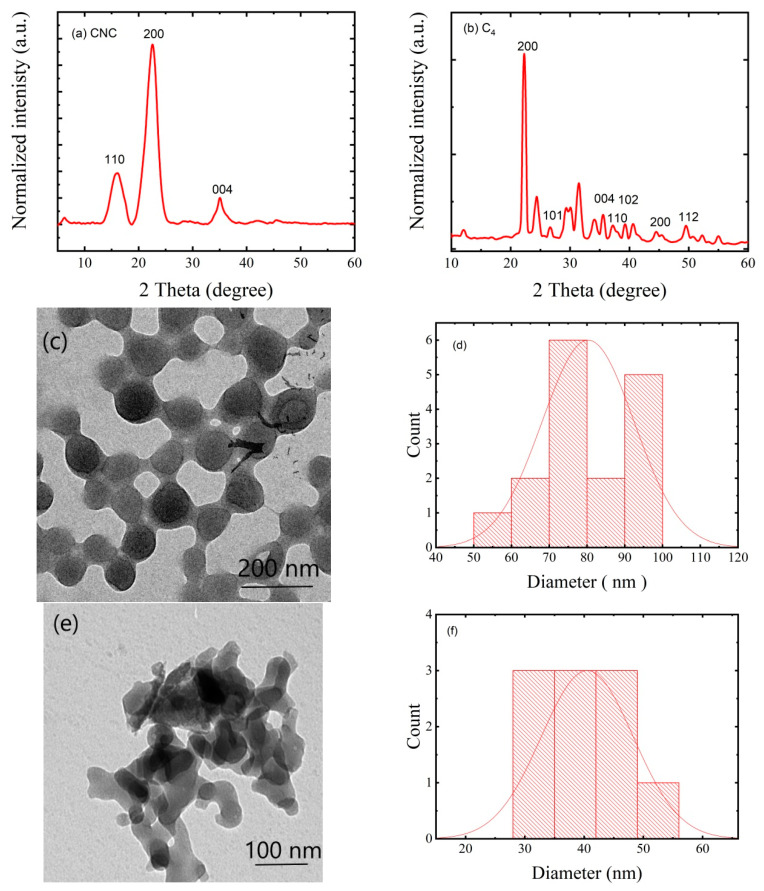
Characteristics of the CNCs and thin film (C_4_). XRD patterns of the (**a**) CNCs and (**b**) thin film (C_4_). (**c**) A TEM image of the CNCs and (**d**) their corresponding particle size distribution. (**e**) A TEM image of ACs and (**f**) their corresponding particle size distribution.

Figure 1a,b show the XRD patterns of the CNCs and thin film (C4). The CNCs showed peaks at 2θ = 15.9°, 22.4°, and 35°, reflecting the (110), (200), and (004) crystalline planes of the normal cellulose I structure, respectively (Figure 1a). The thin film showed 2θ peaks at 22.24°, 26.77°, 35.62°, 37.12°, 39.34°, 44.41°, and 49.66°, reflecting their typical (200), (101), (004), (110), (102), (200), and (112) crystallographic planes, respectively (Figure 1b). TEM images of CNC and AC particles are shown in Figure 1c,e, respectively, with their size ranges shown in Figure 1d (50–80 nm) and Figure 1f (30–50 nm).

### 3.2. Crosslinking and Thin Film Formation

CNC (C_6_H_10_O_5_)_n_ is a natural carbohydrate with several glucose units. Its OH groups and hydrolysis resistance are the most crucial properties for adsorbing cationic dyes and heavy metals from wastewater. CNCs can be enhanced by mixing them with PAA, a hydrophilic, nontoxic, and safe polymer with carboxyl functionalities groups [45].

**Figure 2:**
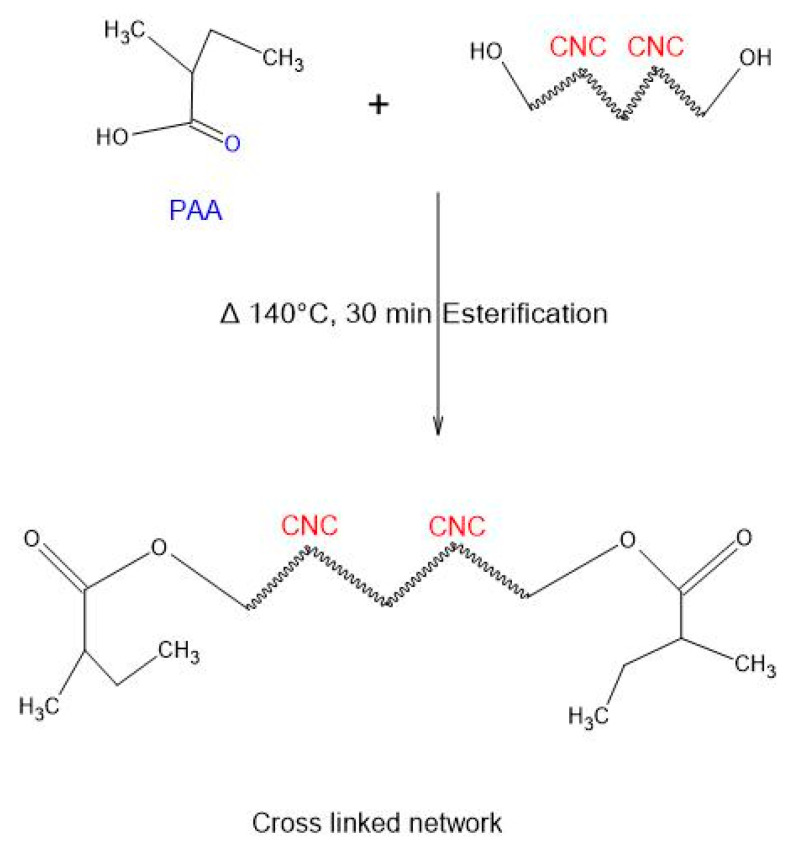
The esterification technique used to create a crosslinked thin film.

Ester production occurs due to the thermally induced crosslinking between the hydroxyl and carboxylic acid groups. The procedure is simple, effective, and quick, producing no hazardous byproducts. Because of the many hydroxyl groups present in CNCs, they interact and crosslink with PAA in the thin film (Figure 2).

**Figure 3:**
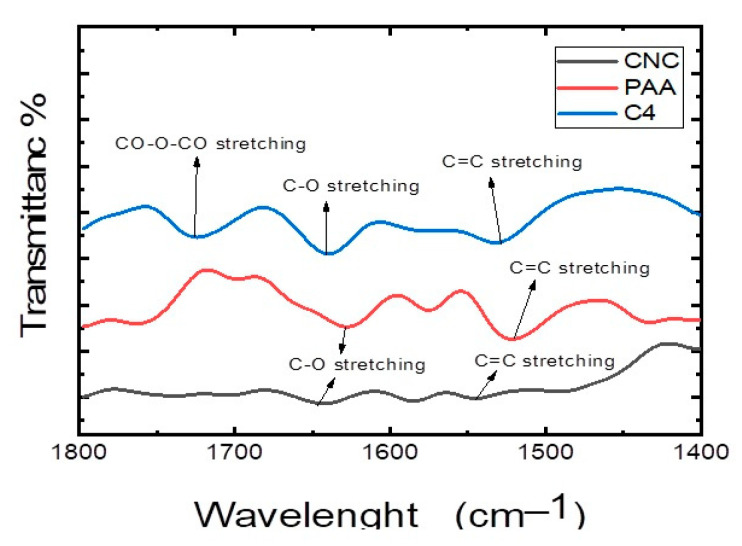
The FTIR spectrum of the CNC/PAA thin film (C_4_).

Crosslinking has been used successfully to improve CNC qualities such as mechanical, thermal, and barrier properties. Furthermore, preparing nanocomposite sites with the highest feasible CNC concentration has significant environmental and economic benefits, emphasizing the need to optimize the CNC:PAA ratio inside the nanocomposite films [46]. Anhydride molecules are produced during the heating of cellulosic materials (CNC and PAA). Figure 3 depicts the creation of cyclic anhydride due to further esterification of the molecule.

The crosslinking between CNC and PAA in the thin film (C4) was examined using FTIR analysis (Figure 3). The CNC spectrum showed peaks for O–H stretching (3700 cm^−1^), a characteristic reflecting hydroxyl groups remaining after the hydrolysis reaction, asymmetric CH_2_ stretching (2987 cm^−1^), a characteristic of distributed cis ranglap bonds, and C=O stretching (1639 cm^−1^). The strong peak centred at 1636.67 cm^−1^ was assigned to the H–O–H stretching mode, a characteristic of H_2_O bending and the C=C bond of the aromatic ring at 1531 cm^−1^ [47]. PAA’s typical IR peaks were detected for O–H stretching (3752 cm^−1^), asymmetric CH_2_ stretching (2900 cm^−1^), C=O stretching (1627 cm^−1^), C=C stretching (1523 cm^−1^), and C–O–C stretching of the polysaccharide skeleton (1000–1200 cm^−1^). The efficient crosslinking of the thin film was confirmed by a new peak at 1724 cm^−1^, which was attributed to the characteristic stretching band of the C=O group linked through ester bonds in the network. This distinctive absorption peak indicated the effective production of the crosslinked thin film.

The thin film’s morphology (C_4_) was evaluated using SEM microscopy, and its elemental composition after MB dye adsorption was examined by energy-dispersive X-ray (EDX) spectroscopy.

**Figure 4:**
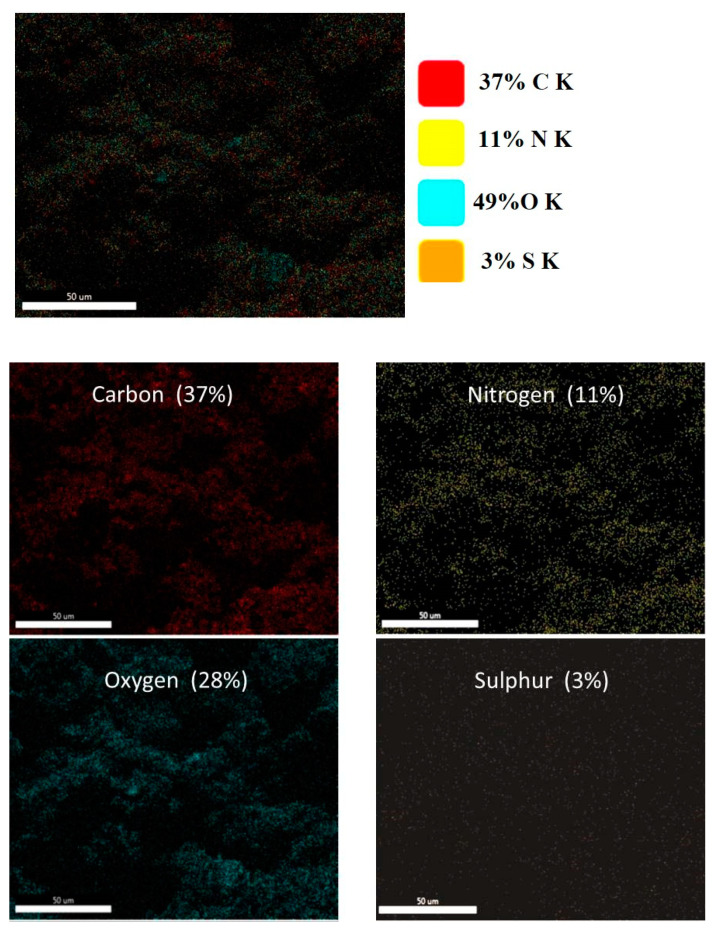
SEM-EDX images of the CNC/PAA/AC thin film (C_4_) after MB dye adsorption. The sulfur and nitrogen distributions correspond to the MB dye.

After MB adsorption, the atomic composition of the C_4_ thin film was 37% carbon, 28% oxygen, 11% nitrogen, and 3% sulfur. Carbon (in red) is the most prevalent element in this composition based on the EDX data, followed by oxygen (Figure 4). Carbon atomic concentrations most likely derive mainly from the CNC, PAA, AC, and the carbon tab of the SEM sample holder. To confirm the adsorption of MB, the sulfur and nitrogen distributions corresponding to the MB dye were obtained using EDX-based element mapping. Sulfur and nitrogen could be used to determine the MB distribution in a thin film.

### 3.3. Crosslinked Thin Film Swelling Effect 

**Figure 5:**
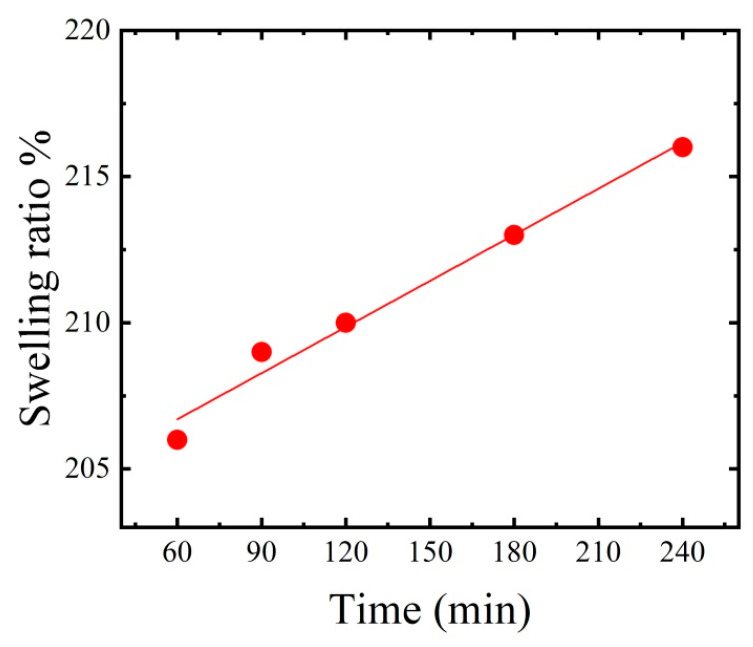
Swelling effect of the thin film (CNC/PAA/AC) composition.

One of the most critical properties of polymer thin films is their ability to swell in water. Water molecules diffuse slowly into the thin film at first, resulting in a bloated thin film. Bulk swelling refers to the swelling of a thin film over an area of 1 mm^2^, whereas microscopic swelling refers to minor dimensional changes. CNC and PAA have negative surface charges, compatible with their high hydrophobicity. The thin film’s swelling capacity is affected by the density of crosslinks, pH, and temperature. The amount of swelling in specific liquids is determined by the thin film’s degree of crosslinking, pore volumes, and functionality. Figure 5 shows 206% swelling of the CNC/PAA thin film after 60 min of immersion in distilled water. Within 60–120 min, the thin film swelled from 206% to 210%. At 240 min, it had reached its maximum value (216%). The CNC/PAA/AC thin films absorb water due to their hydrophilic functional groups (e.g., OH, COOH) linked to the CNC and PAA chains. Surprisingly, thin films with suitable mechanical qualities are favoured for water treatment.

### 3.4. Effect of Thin Film Composition on MB Dye Removal

**Figure 6:**
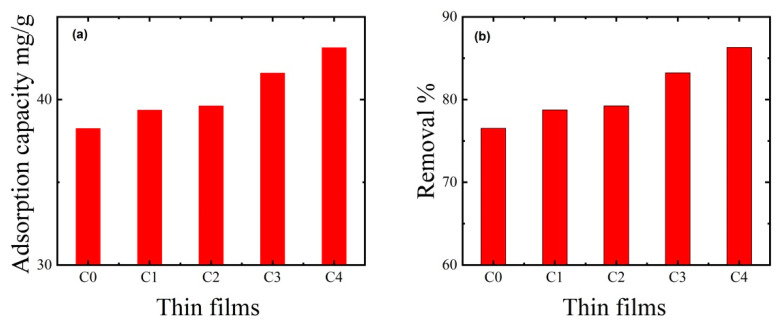
Effect of thin film (PAA/CNC/AC) composition on (**a**) adsorption capacity and (**b**) dye removal at neutral pH (7) and ambient temperature (25 °C). The adsorption experiments used 50 mg of thin film mass, 50 mL of dye solution, an initial MB dye concentration of 50 ppm, a solution pH of 7, an ambient temperature of 25 °C, and a contact time of 2 h.

Adsorption is a common approach for treating dye-contaminated wastewater. The most common issue for sustainable adsorbents is achieving a high dye adsorption capability. Five thin films were produced with varying CNC, PAA, and AC concentrations to obtain a high dye removal capacity (Table 1). The five thin films were evaluated in 50 mL of solutions with a constant initial MB dye concentration (50 mg/L) and pH (7) at a temperature of 25 °C (Table 1. Since the CNC:PAA ratio in C_0_ and C_4_ increased from 0% to 75%, the adsorption capacity increased marginally from 76.53% to 86.30% (Figure 6). It is evident that increasing the CNC:PAA ratio had only a minor effect on dye removal, potentially because it resulted in more negatively charged groups that enabled greater MB dye adsorption. AC particle loading and thin film composition adjustment also helped to improve MB dye removal. With an initial MB concentration of 50 ppm, an initial pH of 7, a temperature of 25 °C, and a contact period of 2 h, the best thin film (C_4_) removed 86.30% of the MB dye and had a dye adsorption capacity of 43.15 mg/g.

### 3.5. Effect of Initial MB Dye Concentration on Its Removal

**Figure 7:**
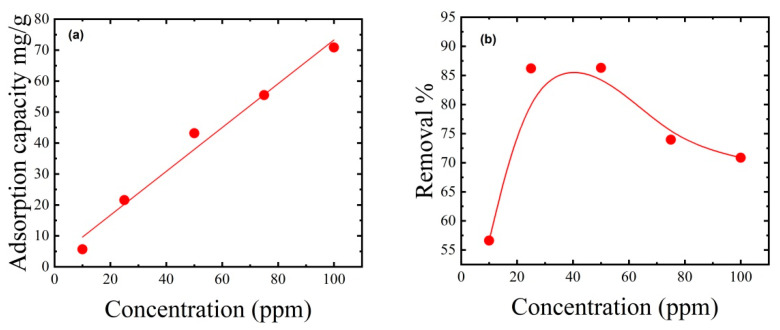
Effect of the initial MB dye concentration on (**a**) adsorption capacity and (**b**) dye removal. The adsorption experiments used 50 mg of thin film mass, 50 mL of dye solution, an initial pH of 7, an ambient temperature of 25 °C, and a contact time of 2 h.

The initial MB dye concentration and thin film mass are crucial in determining dye removal effectiveness from water. For example, dye removal efficiency usually improves with increasing thin film mass since the number of sorption sites increases as thin film mass increases [48]. Therefore, additional MB adsorption experiments were conducted on the best thin film in this study (C_4_). The initial dye concentration ranged from 10 to 100 ppm, while the dye solution volume (50 mL), thin film mass (50 mg), and pH (7) remained constant, and the thin film was agitated at 400 rpm for two hours. Figure 7 shows that as the MB dye concentration increased from 10 to 50 ppm, the adsorption capacity increased from 5.66 to 86.3 mg/g, and dye removal increased from 56.61% to 86.3%. This finding reflects the many adsorption sites available at a low dye concentration of 50 ppm. Increasing the dye concentration from 75 to 100 ppm decreased dye removal from 73.95% to 70.85%, reflecting the formation of two-dimensional cationic dye aggregates, making the adsorption process more difficult and reducing the amount of MB dye removed.

### 3.6. Effect of Contact Time on MB Dye Removal

**Figure 8:**
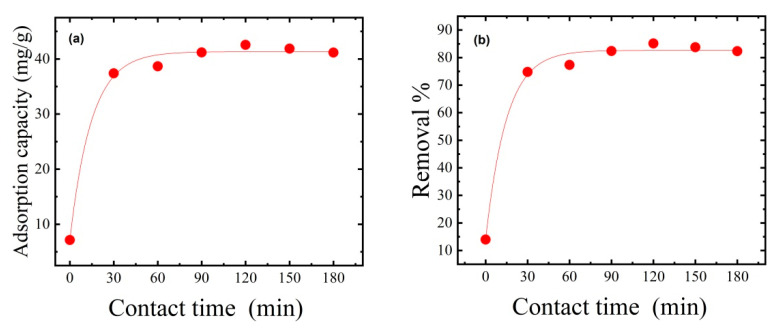
Effect of contact time on (**a**) adsorption capacity and (**b**) percentage dye removal. The adsorption experiments used 50 mg of thin film mass, 50 mL of dye solution, an initial MB dye concentration of 50 ppm, an initial pH of 7, and an ambient temperature of 25 °C.

The dye removal rate increased with contact time due to an increase in the likelihood of the dye adhering to the thin film’s accessible adsorption sites. The time required to reach equilibrium showed the thin film’s maximum adsorption capacity. Adsorption studies were conducted with contact times from 0 to 180 min to study the effects of contact time. The results showed that adsorbed MB dye gradually increased with contact time (Figure 8). The adsorption capacity increases with contact time in the first 120 min until an equilibrium is established [49]. Increasing contact time from 0 to 120 min increased adsorption capacity from 7.14 to 42.57 mg/g and dye removal from 14% to 85.14%. These findings are consistent with Somsesta et al. [50], who created AC/cellulose composite sheets for MB dye removal and investigated the effect of exposure time. They found that as contact time increased, the adsorption capacity increased until equilibrium was reached.

### 3.7. Effect of Temperature on MB Dye Removal

**Figure 9:**
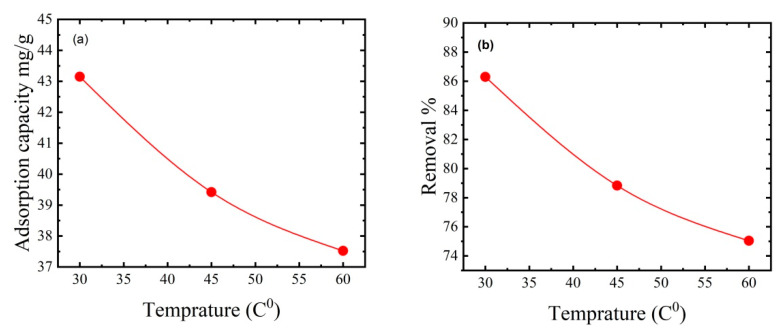
Effect of temperature on (**a**) adsorption capacity and (**b**) percentage dye removal. The adsorption experiments used 50 mg of thin film mass, 50 mL of dye solution, an initial MB concentration of 50 ppm, an initial pH of 7, and a contact time of 2 h.

The dye solution’s temperature is one of the most important factors influencing adsorption capacity. Adsorption tests were conducted at 25 °C, 40 °C, and 60 °C to investigate the effects of temperature. Figure 9 shows that as temperature increased, MB adsorption decreased from 43.15 to 37.52 mg/g, while dye removal decreased from 86.3% to 75.04%. The decreased MB dye removal with increasing temperature may reflect the weakening of the adsorption forces between the MB dye molecules and the thin film’s active sites and between nearby molecules in the adsorbed phases [51]. These findings are consistent with those of Mohammed et al. [52], who developed CNC/alginate hydrogel beads and investigated the influence of temperature on MB dye removal. They found that increasing the temperature from 25 °C to 55 °C reduced dye removal efficacy from 77% to 64%.

### 3.8. Effect of pH on MB Dye Removal

**Figure 10:**
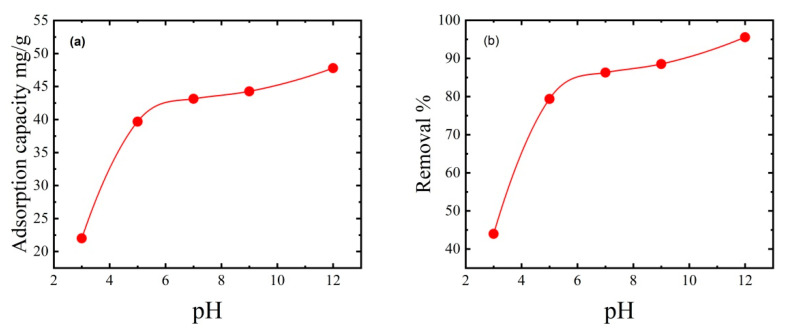
Effect of initial pH on (**a**) adsorption capacity and (**b**) percentage dye removal. The adsorption experiments used 50 mg of thin film mass, 50 mL of dye solution, an initial MB dye concentration of 50 ppm, an ambient temperature of 25 °C, and a contact time of 2 h.

The pH of the dye solution regulates the number of electrostatic charges transferred from ionized dye molecules, influencing the dye adsorption capacity [53]. Cationic dye adsorption is best at high pH, whereas anionic dye adsorption is less efficient. Because the electrostatic attraction between the positively charged MB dye and the thin film surface increases with increasing pH, the adsorption capacity increases [54]. The ionization of the thin film’s functional surface groups and the ionization of MB dye molecules are affected by pH changes. Experiments were performed with 50 mg of thin film support mass, 50 mL of dye solution, an initial MB dye concentration of 50 ppm, and an ambient temperature of 25 °C to explore the effects of pH. The solution was adjusted to an initial pH of 3, 5, 7, 9, and 12 with dilute HCl and NaOH. Figure 10 shows that at a pH of 3, the adsorption capacity was 22 mg/g, and the removal efficiency was 44%, while at a pH of 7, dye removal reached up to 86.3%. Increasing the initial pH from 7 to 12 achieved an excellent dye adsorption capacity of 47.78 mg/g and a dye removal efficiency of 95.56%. Therefore, pH values >8 favoured MB dye adsorption by the prepared thin film. These results agree with Oyewo et al. [55], who prepared sawdust-based CNCs containing zinc oxide nanoparticles and investigated the influence of the initial pH. Changing the initial pH from 2 to 10 increased dye removal from 60% to 90% [56].

### 3.9. Adsorption Kinetics and Thermodynamic Isotherm

**Figure 11:**
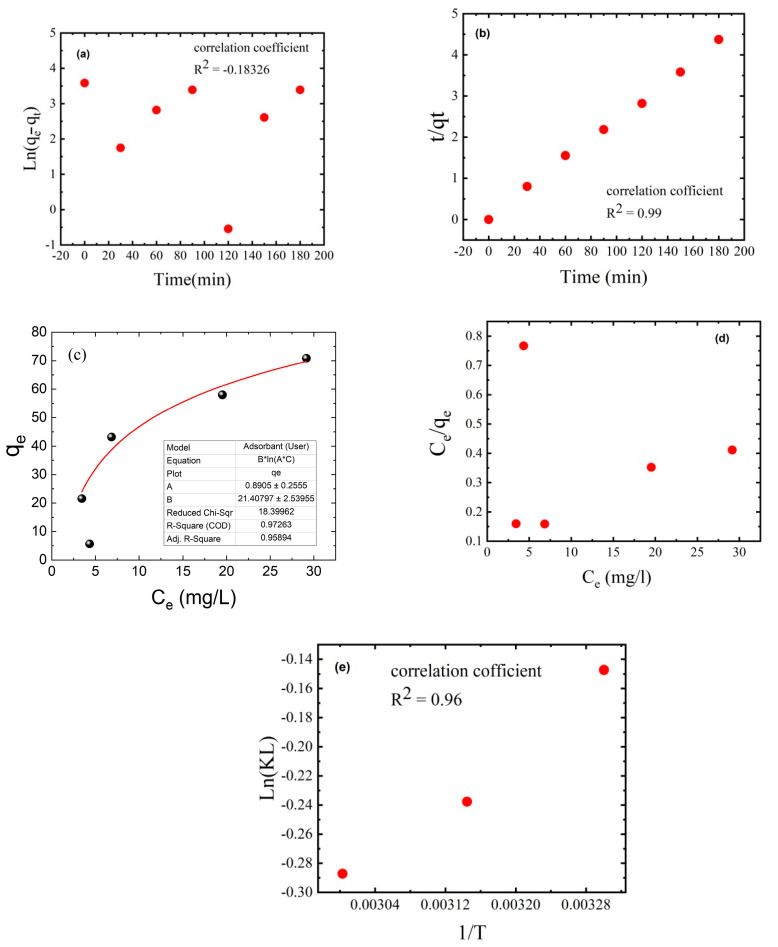
Adsorption experiments of MB dye fitting with (**a**) pseudo-first-order and (**b**) pseudo-second-order kinetics at an ambient temperature of 25 °C, and (**c**) Freundlich and (**d**) Langmuir isotherms at 25 °C (298 K), 45 °C (318 K), and 60 °C (333 K). ineticsa (**e**) dsorption curce The adsorption experiments used 50 mg of thin film mass, 50 mL of dye solution, an initial MB dye concentration of 50 ppm, an initial pH of 7, and a contact time of 2 h.

Adsorption kinetics was used to determine MB dye adsorption capabilities as a function of time at a constant dye concentration. Adsorption kinetic studies were performed with a C_4_ thin film mass of 50 mg, an initial MB dye concentration of 50 ppm in 50 mL of dye solution, an initial pH of 7, a temperature of 25 °C, and a contact time of 2 h. The experimentally determined adsorption capacity was 43.15 mg/g. Pseudo-first-order and pseudo-second-order kinetic models were used to fit the experimental kinetic data (Figure 11a,b). The adsorption capacity at equilibrium time (*q*_e_ = 42.13 mg/g), correlation coefficient (R^2^ = 0.998), and reaction constant (*k*_2_ = 0.011184513 g/mg.min) obtained with the pseudo-second-order model were close to those obtained with the other published models. However, the adsorption capacity at equilibrium time (*q*_e_ = 14.39 mg/g), correlation coefficient (R^2^ = −0.180), and reaction constant (*k*_1_ = −1.47 × 10^−5^ min^−1^) obtained with the pseudo-first-order model were far from those obtained with the other published models. These findings suggest that the adsorption of the investigated adsorbates occurs via a chemisorption process and that the pseudo-second-order mechanism predominates in the MB dye adsorption processes [57]. Fitting the experimental data gathered at the equilibrium time point with Langmuir and Freundlich isotherm models yielded thermodynamic adsorption isotherms. These models demonstrate the relationship between adsorption capacity and equilibrium concentration at a constant temperature [58]. According to the Langmuir adsorption model, a saturated monolayer of dye molecules on the adsorbent surface corresponds to the maximum restricted absorption [59]. The Freundlich adsorption isotherm assumes that adsorption occurs on a heterogeneous surface via a multilayer adsorption mechanism and that the amount adsorbed increases as the dye concentration increases [60,61].

The linear forms of the studied isothermal models are shown in Equations (7) and (8):

(7) $$\frac{C_{e}}{q_{e}}=\frac{K_{s}}{q_{\max}}+\frac{C_{e}}{q_{\max}}$$

(8) $$\log q_{e}=\frac{1}{n}\log C_{e}+\log p$$

where *q*_e_ is the amount of MB dye adsorbed at equilibrium (in mg/g), *C_e_* is the dye equilibrium concentration in solution (in mg/L), *q*_m_ is the maximum adsorption capacity of the thin film of a given mass, and *k*_L_ and *k*_F_ are the Langmuir and Freundlich constants, respectively. According to the correlation coefficient (R^2^), the Langmuir isotherm model best described the MB dye adsorption process (Figure 11d). The MB dye’s theoretical maximum adsorption amount (*q*_max_), obtained with the Langmuir isotherm model, was 76.6 mg/g, close to the experimental values (76.5 mg/g). The better fitting of the Freundlich isotherm model means that MB dye molecules cover the membrane surface by multilayer adsorption on heterogeneous membrane sites. Figure 9e shows that the effect of temperature on MB dye adsorption at 25 °C (298 K), 45 °C (318 K), and 60 °C (333 K), and the thermodynamic parameters including ΔS, ΔH, and ΔG, were calculated based on the adsorption capacity *q*_e_ (in mg/g) and concentration *C*_e_ (in mol/L). The results showed that as the temperature increased from 25 °C (298 K) to 45 °C (318 K) to 60 °C (333 K), ΔG increased from −0.37 to 0.63 to 0.79 kJ/mol, respectively. This finding indicates that high temperature is not beneficial for spontaneous MB dye adsorption, similar to the experimental results in Figure 9. The ΔH value for MB dye adsorption was −3.93 kJ/mol, indicating exothermic physical adsorption of MB dye molecules on the C_4_ thin film. The negative ΔS of −14.23 JK^−1^/mol indicated that randomness at the interface between the thin film and the MB dye solution decreased during adsorption [62].

## 4. Conclusions

Thin films composed of CNC, PAA, and AC have received considerable attention due to their unique properties, including high specific surface area, low weight, nontoxicity, chemical reactivity for surface modification, biocompatibility, high hydrophilicity, and environmental friendliness. This study produced an environmentally friendly adsorbent thin film composed of CNC, PAA, and AC to remove azo (MB) dyes. The importance of this study lies in how to extract nanomaterials such as cellulose nanocrystals from agricultural waste such as sugarcane bagasse and use it with certain additives (PAA, AC) to adsorb the largest possible amount of methylene blue dye. The effect of crosslinking on increasing the thin film’s hydrolytic stability was shown in the swelling investigation. The C_4_ thin film formulation (64% CNC, 16% PAA, and 20% AC) showed excellent adsorption with an alkaline pH, increasing from 86.3% at neutral pH to 95.56% at pH 7–12. This finding indicates that at pH values > 7, the surfaces of the thin film’s components (CNCs and PAA) were negatively charged due to OH^−^ ions. Therefore, electrostatic attraction forces rapidly adsorb the MB dye cations on these surfaces. Consequently, pH values > 7 are beneficial for MB dye adsorption on the produced thin film. Adsorption studies showed that the produced thin films effectively removed MB dye from aqueous solutions. Adsorption equilibrium was achieved in about two hours. Furthermore, temperature effects on adsorption uptake were used to calculate its thermodynamic parameters. These findings show that the C_4_ thin film could be a good adsorbent for removing dyes from contaminated water sources, which is different than other similar work present in the literature in the following ways: Firstly, the study used the extraction of cellulose nanocrystals from bagasse, which is considered an agricultural waste. Secondly, it used different concentrations of cellulose nanocrystals to determine the extent of its adsorption of the dye. Thirdly, it employed the addition of activated carbon to this composition. Fourthly, the results indicated the high adsorption of methylene blue using this composite. Notably, it has a higher adsorption capacity for the MB dye than other published adsorbents (Table 2).

## Figures and Tables

**Table 1:** Five thin films were prepared using different CNC, PAA, and AC concentrations. The adsorption experiments used 50 mg of the thin film, an initial MB concentration of 50 ppm, a solution pH of 7, a room temperature of 25 °C, and a contact time of 2 h.

Thin Film Formulation	Thin Film Composition			Dye Removal Efficiency	
	CNC	PAA	AC	Removal Capacity	Removal Percentage
C_0_	0.0%	80.0%	20%	38.26 mg/g	76.53%
C_1_	40.0%	40.0%	20%	39.37 mg/g	78.75%
C_2_	53.30%	26.65%	20%	39.62 mg/g	79.24%
C_3_	60%	20%	20%	41.61 mg/g	83.22%
C_4_	64%	16.0%	20%	43.15 mg/g	86.30%

**Table 2:** Comparison of monolayer adsorption capacities for the MB dye of reported bio-sorbents.

Adsorbent	Maximum Adsorption Capacity (mg/g)	Reference
**Rice husk**	19.77	[63]
**Wheat straw**	3.82	[64]
**Milled sugarcane**	9.90	[65]
**Activated carbon**	9.81	[66]
**Almond shell-AC**	1.33	[67]
**Microcrystalline cellulose**	12.85	[68]
**TiO2/Poly(acrylamide-co-acrylic acid)**	21.3	[69]
**CNC, PAA, and AC**	43.15	Our work

## Data Availability

Detailed data supporting this study’s findings are included in the article. The originally collected data are available upon reasonable request from the corresponding author.
